# The Six-Minute Stepper Test Is Valid to Evaluate Functional Capacity in Hospitalized Patients With Exacerbated COPD

**DOI:** 10.3389/fphys.2022.853434

**Published:** 2022-06-24

**Authors:** Diego Britto Ribeiro, Aline Carleto Terrazas, Wellington Pereira Yamaguti

**Affiliations:** Hospital Sírio-Libanês, Rehabilitation Service, São Paulo, Brazil

**Keywords:** COPD, exacerbation, physical activity, exercise capacity, dynamic hyperinflation

## Abstract

**Background:** The six-minute stepper test (6MST) is a self-paced test considered a valid tool to assess functional capacity in stable COPD patients. However, a high floor effect, where a large proportion of participants reach the minimum score when using the measurement instrument, might compromise the test validity in the hospital setting. Therefore, this study aimed at verifying the concurrent validity of 6MST in hospitalized patients with acute exacerbation of COPD (AECOPD).

**Methods:** A cross-sectional study was conducted in a tertiary hospital. Patients who were hospitalized due to AECOPD were considered for inclusion. On the first day, when patients reached minimum clinical criteria considered as the use of non-invasive ventilation less than 2 h for 6 h/period, dyspnea at rest less than 7 (very severe) on the modified Borg scale, a respiratory rate less than 25 breaths per minute, oxygen pulse saturation greater than 88% (considering use of supplemental oxygen) and absence of paradoxical breathing pattern, they underwent a lung function evaluation and answered three questionnaires: Chronic Respiratory Questionnaire (CRQ), Modified Medical Research Council Dyspnea Scale (MMRC), and COPD Assessment Test (CAT). Then, on two consecutive days, patients performed 6MST or six-minute walk test (6MWT), in random order. Each test was performed twice, and the best performance was recorded. Also, the patient’s severity was classified according to the BODE index. Inspiratory capacity measurements were performed before and after each test execution.

**Results:** Sixteen patients (69.4 ± 11.4 years) with a mean FEV₁ of 49.4 ± 9.9% predicted were included (9 females). There was a strong correlation of the performance in 6MST (number of cycles) with 6MWT (distance walked in meters) in absolute values (r = 0.87, *p* < 0.001) as well as with the percentage of predicted normal 6MWT (r = 0.86, *p* < 0.001). There was a strong correlation between the performance in 6MST with the dynamic hyperinflation (r = 0.72, *p* = 0.002) and a moderate correlation between 6MST with the percentage of reduction of inspiratory capacity (r = 0.68, *p* = 0.004). We also identified that 6MST showed moderate negative correlations with CAT (r = −0.62, *p* = 0.01) and BODE index (r = −0.59, *p* = 0.01).

**Conclusion:** It could be concluded that 6MST is valid for evaluating functional capacity in hospitalized patients with exacerbated COPD.

## Introduction

COPD is one of the leading causes of morbidity and mortality worldwide, resulting in a substantial and growing economic and social impact (Lopez et al., 2006). Exacerbations of COPD are directly associated with worsening quality of life (Vestbo et al., 2013), accelerated decline in lung function (Garcia-Aymerich et al., 2011), reduced exercise capacity (Pitta et al., 2006), reduced daily living activities (Pitta et al., 2006), a significant increase in mortality (especially in patients requiring hospitalization) (Garcia-Aymerich et al., 2011), and increased socioeconomic costs (Vestbo et al., 2013). Exacerbated COPD patients have an amplification of the reduced exercise capacity characteristics of the disease (Pitta et al., 2006). In addition to ventilatory impairment, which is the main limitation of exercise capacity, other factors can also contribute to this reduction, such as peripheral muscle weakness (Choudhury et al., 2014). Several mechanisms can contribute to the skeletal muscle weakness found during acute exacerbation (Spruit et al., 2003; Pitta et al., 2006), such as the presence of systemic inflammation (Spruit et al., 2003), nutritional changes (Creutzberg et al., 2000), administration of oral corticosteroids (Decramer et al., 1994), and a sedentary lifestyle (Pitta et al., 2006).

Even with the appropriate adjustment of pharmacological therapy, the presence of high morbidity and mortality, as well as the persistent intolerance to physical exercise that occurs in patients with COPD, justify the need for research on new treatment strategies, such as regular and continuous physical training, based mainly on the patient’s functional capacity. Furthermore, it is known that functional capacity is impaired in this profile of patients, and may present progressive worsening during hospitalization, thus, the correct assessment of functionality becomes essential, contributing positively to the planning of individually designed therapeutic strategies, optimization of prescription of exercises, in addition to monitoring the evolution of their functionality and clinical condition, as well as the responses and progress of rehabilitation programs in hospitalized patients. An established and widely used way to assess the functional capacity of these patients is the 6-min walk test (6MWT). However, although the 6MWT is a simple field test, well adapted and validated in several populations, a low-cost and widely feasible test, it has some disadvantages. Perhaps the main one is the need for a corridor to perform the walk with at least 30 m, to represent a valid test, as recommended by the American Thoracic Society and European Respiratory Society (Holland et al., 2014). Thus, to circumvent this 6MWT disadvantage, especially in the hospital environment, the assessment of functional capacity can be performed using other tests, such as the step test (José and Dal Corso, 2016), the Four-meter gait speed (Kon et al., 2013), the Timed “Up and Go” (Johnston et al., 2017), as well as the six-minute stepper test (6MST) (Borel et al., 2010).

The 6MST uses a stepper, a portable device that simulates the climbing of steps, moving with the individual’s action, eliminating the need for an extensive corridor to perform the assessment. The 6MST is a self-paced test, like the 6MWT, and its primary outcome is the number of cycles completed in 6 minutes. The 6MST is considered a feasible technique for assessment of functional capacity in patients with different diseases such as COPD (Coquart et al., 2014), interstitial lung diseases (Delourme et al., 2012), older adults (Jones et al., 2017), home-based pulmonary rehabilitation (Grosbois et al., 2019a; Grosbois et al., 2020), asthma (Grosbois et al., 2019b), fibrotic idiopathic interstitial pneumonia (Wallaert et al., 2019), and chemotherapy-treated patients with thoracic cancers (Olivier et al., 2018), as an alternative to the 6MWT. Furthermore, in a population of hospitalized and healthy older people, the 6MST showed convergent validity with the functional variables used to diagnose sarcopenia (Francisco et al., 2020). 6MST is a submaximal test, reproducible and well-tolerated assessment tool. In addition, it is an inexpensive and portable method to assess exercise tolerance in patients with stable COPD (Borel et al., 2010). In a previous study, 6MST was applied to circumvent the environmental restrictions of the 6MWT, and significant correlations were observed between the 6MST and the 6MWT in oxygen consumption and heart rate (Borel et al., 2010). That same study also suggested discriminative properties of the test, as it found significantly higher performance in healthy individuals compared to patients with COPD.

Furthermore, another study was also able to demonstrate the sensitivity of the 6MST to detect improvement in functional capacity after pulmonary rehabilitation in patients with COPD (Coquart et al., 2014). In addition, another relevant characteristic of 6MST is that it allows the prescription of training for patients with COPD (Bonnevie et al., 2017). Although the 6MST has been validated in stable patients, the possibility of a high floor effect could compromise the validity of this tool in the hospital setting, that is, individuals who reach the minimum score when using the 6MST due to the possible limitations of their clinical condition. Thus, this study aimed at verifying the concurrent validity of 6MST to assess the functional capacity in hospitalized patients with acute exacerbation of COPD (AECOPD).

Our study was based on the COnsensus-based Standards for the selection of health Measurement INstruments (COSMIN) and its checklist (Mokkink et al., 2010), for analysis of the methodological quality of the concurrent validation assessment (COSMIN box H. Criterion Validity). Criterion Validity is the degree to which the scores of a measuring instrument are an adequate reflection of a gold standard (Scholtes et al., 2011), in our case, the 6MWT.

## Patients and Methods

### Ethics and Participants

The sample was obtained consecutively, recruiting patients of both sexes admitted to the Hospital Sírio-Libanês for treatment of AECOPD. The study included individuals who met the following criteria: 1) patients with a previous medical diagnosis of COPD before hospitalization; 2) diagnosis of exacerbated COPD classified as level II (Celli et al., 2004b), 3) absence of cognitive or motor deficit that limited the execution of the tests; 4) absence of previous cardiovascular disease; 5) no previous thoracoabdominal surgery within 1 month; 6) body mass index (BMI) < 30 kg/m^2^; and 7) no use of vasoactive drugs. The exclusion criteria were considered: 1) inability to perform the evaluations within the criteria of technical acceptability and 2) cardiorespiratory instability during the tests (severe dyspnea, arrhythmias, angina, elevated heart rate above 80% of maximum heart rate, and oxygen pulse saturation below 88% refractory to oxygen supplementation). The study was previously approved by the research ethics committee of the Hospital Sírio-Libanês (approval protocol 3.432.823) and written informed consent was provided by all participants.

### Study Design and Experimental Procedures

In the cross-sectional design of this study, the patients underwent an evaluation protocol performed on two consecutive days. The protocol was applied from the moment that the patients had the following minimum clinical criteria: use of non-invasive ventilation less than 2 h per 6-h/period, dyspnea at rest less than 7 (very intense) on the modified Borg scale, respiratory rate less than 25 incursions per minute, oxygen pulse saturation greater than 88% (considering supplemental oxygen use) and absence of paradoxical breathing pattern. On the first day of the evaluation, the subjects answered three specific evaluation questionnaires related to lung disease: 1) the Chronic Respiratory Questionnaire (CRQ), 2) the modified Dyspnea Scale of the Medical Research Council (MMRC), and 3) the COPD Assessment Test (CAT). In addition, anthropometry, pulmonary function tests, and two evaluations of 6MST and 6MWT (in randomized order by closed envelopes) were performed. Thirty minutes after the execution of the first test, the same test selected was conducted again. On the second day, individuals underwent the other exercise tolerance test (6MST or 6MWT, as randomized), which was also repeated after 30 min. Each test was carried out twice and the best performance was recorded. Also, the patient’s severity was classified according to the BODE index. Inspiratory capacity measurements were obtained before and after each test execution.

The pulmonary function test was performed using a portable spirometer (Koko pulmonary function testing model; nSpire Health Company, Longmont, CO, United States), previously calibrated according to the methods and criteria recommended by the American Thoracic Society (Miller et al., 2005). At least three acceptable maneuvers and two repeatable maneuvers were performed. The highest values obtained for each of the spirometric variables were considered, which were expressed in absolute and in the percentage of the expected values of normality (Pereira et al., 2007). Post-bronchodilator spirometry measurements were performed to assess the response to drug therapy. Forced vital capacity (FVC), forced expiratory volume in the first second (FEV₁), FEV₁/FVC ratio, vital capacity (VC), and inspiratory capacity (IC) were assessed.

The CRQ has been widely used in the analysis of the health status of patients with COPD (Moreira et al., 2009). This questionnaire contains 20 questions, divided into four domains: dyspnea, fatigue, emotional function, and self-control. A higher score achieved is associated with a better quality of life for the subject.

A widely used tool to assess the effect of dyspnea on activities of daily living is the MMRC scale. It consists of a 5-item questionnaire in which patients categorize their degree of disability, reflecting how dyspnea affects their mobility (Wedzicha et al., 1998; Bestall et al., 1999). The patients report their subjective degree of dyspnea, choosing a value between 0 and 4. Lower scores in the MMRC are associated with less impairment of activities of daily living related to dyspnea.

The CAT questionnaire was used to assess the clinical impact of COPD symptoms. This tool has the characteristics of being a short and simple instrument to quantify the impact of COPD symptoms on clinical practice, assist in assessing health status, and facilitate communication between patients and the health professional (Jones et al., 2012). This questionnaire has been validated for the Brazilian population with COPD (Silva et al., 2013). It consists of eight items: cough, phlegm, chest tightness, shortness of breath, limitations in-home activities, confidence in leaving home, sleep, and energy. Results vary according to the score range obtained, classified as follows concerning clinical impact: 6–10 points, mild; 11–20, moderate; 21–30, severe; and 31–40, very severe.

The BODE index serves as a predictor of mortality risk that assesses individuals with COPD systemically (Celli et al., 2004a; Araujo and Holanda, 2010). Furthermore, it is also used to classify the severity of COPD, determine the risk of hospitalizations due to exacerbation (Ong et al., 2005), and predict response to pulmonary rehabilitation programs (Cote and Celli, 2005). Its assessment includes the body mass index (BMI), the degree of obstruction to the expiratory flow through FEV₁, the perception of dyspnea using the MMRC scale, and the exercise tolerance, assessed by the performance in the 6MWT. However, its greater use and importance stems from the fact that it does not evaluate the degree of airway obstruction in isolation. It analyzes COPD’s respiratory and systemic manifestations and can better characterize and predict outcomes in this population (Da Silva et al., 2013).

The 6MWT was performed in a flat corridor 30 m long and 1.5 m wide, previously marked. The individuals were instructed and encouraged to walk as far as possible for 6 minutes, using standardized incentive phrases every minute, as recommended by the American Thoracic Society (ATS) and European Respiratory Society (ERS) (Holland et al., 2014). The 6MWT was performed twice on the same day, with an interval of 30 min between tests. For the analysis, the longest distance presented between the two tests was used. The predicted values were obtained from the reference equation proposed by Britto and colleagues (Britto et al., 2013). Inspiratory capacity (IC), oxygen pulse saturation, respiratory rate (RR), heart rate (HR), symptoms of dyspnea and lower limb fatigue (verified by the modified Borg scale), and blood pressure measurements were checked at rest and immediately after the test. Pulmonary hyperinflation, defined as the difference between the IC measured in the pre-and post-test period, was considered present when a 10% and/or 150 ml reduction in the post-test IC was demonstrated concerning the pre-test IC (O'Donnell et al., 2001; Colucci et al., 2010). All measurements in both tests (6MWT and 6MST), were evaluated with the patient seated upright, with feet flat on the floor and legs uncrossed. Measurements at the end of the test were performed as soon as the test ended and the patient assumed the sitting position. Two evaluators performed data collection and measurements. The inspiratory capacity (IC) was assessed using a portable spirometer (Koko pulmonary function testing model; nSpire Health Company, Longmont, CO, United States). The IC measurement maneuver was performed after the stabilization of the end-expiratory lung volume verified by the equipment. The patient was encouraged to perform a maximal voluntary inspiratory maneuver for TLC both during the evaluation at rest and at the end of the exercise. The oxygen pulse saturation and heart rate (HR) were assessed using a portable pulse oximeter (pulse oximeter Nonin; Nonin Medical Inc., Plymouth, MN, United States) and the blood pressure measurements were checked using a sphygmomanometer (sphygmomanometer Omron, Omron Healthcare Brasil, Jundiaí, SP, Brazil). One evaluator performed respiratory rate counting and questioned the patient about dyspnea and lower limb fatigue symptoms. The other evaluator assessed the oxygen pulse saturation, heart rate, blood pressure, and inspiratory capacity measurements.

**FIGURE 1:**
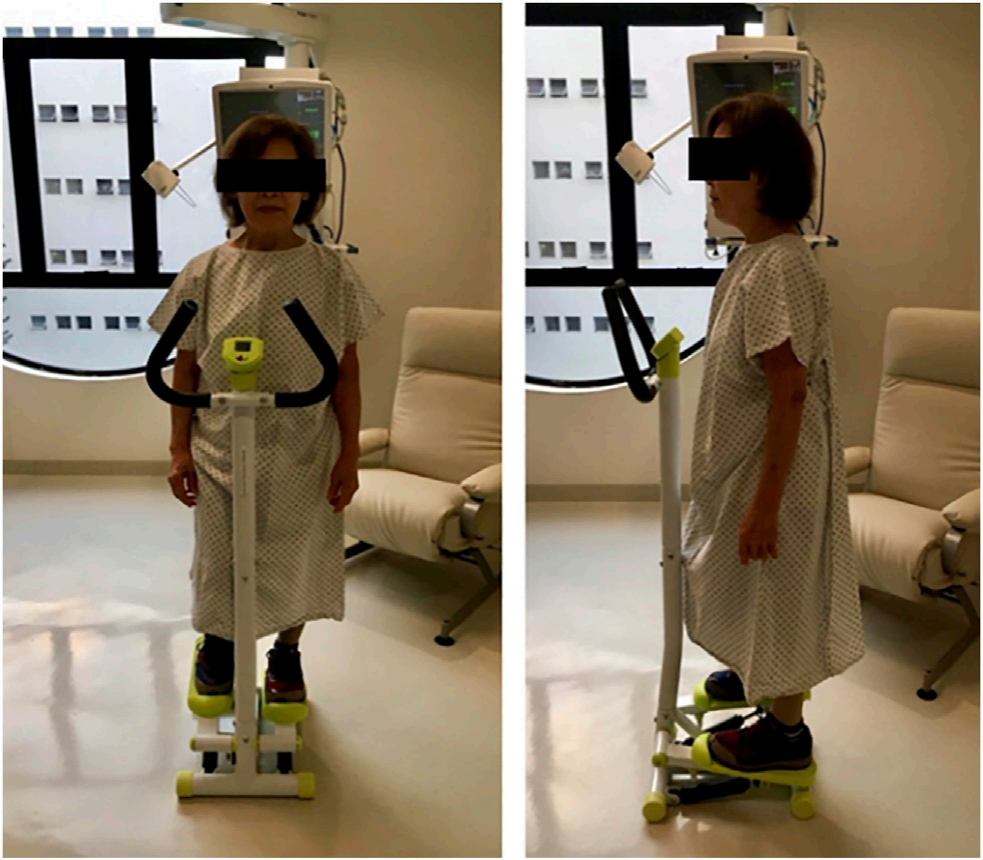
Pictures of the stepper and position adopted during the six-minute stepper test.

The 6MST, using the stepper (Mini Stepper, Mor, Santa Cruz do Sul, RS, Brazil), was performed in an isolated room to avoid any stimulus that could influence the performance of the activity. The stepper used had a support rod to allow the patients to support themselves when they were unbalanced or exhausted. The initial position of the stepper was as follows: right or left foot, according to the individual’s choice, in the elevated position and the other foot in the lower position, with the arms along the body (Figure 1).

The adjustment of the height of the elevated position of the stepper was 20 cm. The test was performed after the patient’s familiarization with the equipment, remaining at rest in the initial position for a period of up to 2 minutes, if necessary. The 6MST followed the same ATS/ERS recommendations for the 6MWT, using the same standardized incentive phrases every minute. Patients were instructed and encouraged to perform the full cycle of the device as many times as possible in 6 minutes.

The full cycle is defined as the return to the starting position (Borel et al., 2010). The value provided by the device’s digital display was used to count the total number of cycles performed in the stepper. The equipment display was positioned opposite the patient, making it impossible for the participant to see the step count. 6MST was performed twice on the same day, with an interval of 30 min between tests. The number of full cycles performed in the tests was used for the analysis. As with the 6MWT, measurements of IC, oxygen pulse saturation, respiratory rate, heart rate, symptoms of dyspnea and lower limb fatigue, and blood pressure were checked at the same time as the test was performed.

### Statistical Analyses

The GraphPad Prism 6 Statistical Package (GraphPad Software, San Diego, CA, United States) was used for the statistical analysis of the data, presented by the mean and standard deviation (SD). The normality of the data was assessed using the Shapiro Wilk test. To evaluate the correlation of the performance in 6MST with the performance in 6MWT, dynamic hyperinflation, questionnaires, and specific index associated with lung disease (MMRC, BODE, CAT, and CRQ), spirometric variables (FEV₁, FEV₁/FVC, FVC, and baseline IC), and variations of vital signs (oxygen pulse saturation, HR, systolic and diastolic blood pressure, and RR) and the perception of dyspnea and fatigue of lower limbs before and after testing, and between the variations magnitude of vital signs and perception of dyspnea and fatigue of lower limbs from rest during the 6MST and 6MWT, Pearson’s correlation test (parametric data) or Spearman’s correlation test (nonparametric data) were used. The magnitude of the correlations was based on Munro’s classification (Munro, 2001): none or small, from 0 to 0.25; weak from 0.26 to 0.49; moderate, from 0.50 to 0.69; strong, from 0.70 to 0.89; very strong, from 0.90 to 1.00. The performances of the two 6MST tests were compared for repeatability analysis using the paired Student’s t-test (parametric data). Repeatability is considered the variation of the response depending on the same measuring device and evaluator; therefore, repeatability refers to the performance of serial measurements under the same conditions, performed over a short time, by the same evaluator using the same equipment. For all tests, a *p* < 0.05 was considered statistically significant. According to the COSMIN guidelines, it is possible to perform sample size calculations for expected correlations between measures in validity studies. The 6MST and 6MWT instruments have continuous scores. Therefore, the preferred method is the presentation of the correlation coefficient. This correlation should preferably be above 0.70 (COSMIN box H-10). Based on the results of a previous study (Pichon et al., 2016) which found a significant correlation (r = 0.72) between the number of steps during 6MST and the 6MWT pre-pulmonary rehabilitation, and estimating a similar effect, using an error of 5% (power test time 90%), the need to include 16 patients in the study was calculated (COSMIN box H-3).

## Results

**FIGURE 2:**
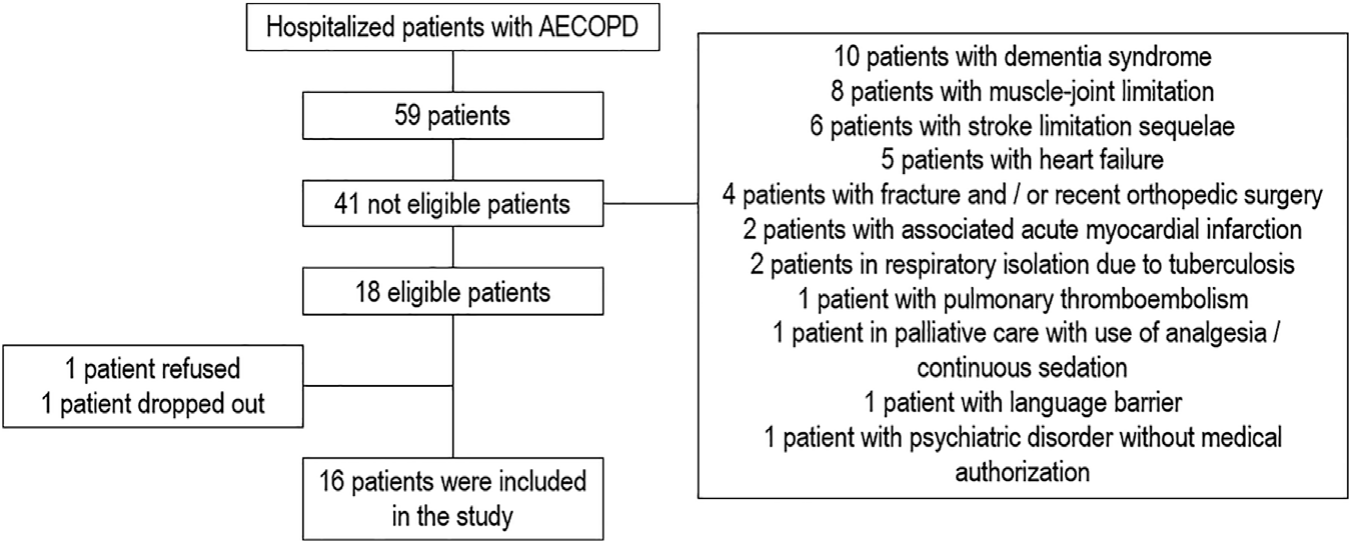
Screening flowchart for hospitalized patients with AECOPD. Abbreviations: AECOPD, acute exacerbation of COPD.

A total of 59 hospitalized patients with a diagnosis of exacerbated COPD were screened, among which 41 patients did not meet the inclusion criteria. In total, 18 patients met the inclusion criteria; however, 1 patient refused, and 1 patient dropped out on the second day of evaluation (Figure 2) (COSMIN box H-5). Thus, a total of 16 patients were included in the study, being 9 female (56.25%) and 7 male (43.75%), with a mean age of 69.38 ± 11.42 years and FEV₁ of 49.38 ± 9.86% of the predicted. According to the GOLD classification, 8 patients (50%) were categorized as GOLD II, and 8 patients (50%) as GOLD III. These results are described in Table 1. The results of the performance average in the first, second, and the best 6MWT and 6MST; and physiological and perceptual parameters at rest and the end of the best tests performed are described in Tables 2, 3, respectively. There was no data loss for any variables (COSMIN box H-1 and H-2).

**TABLE 1:** Characterization of the study participants (*n* = 16).

Demographic and anthropometric data	
Age (years)	69.4 ± 11.4
Sex (M/F)	7/9
BMI (kg/m^2^)	23.7 ± 4.7
Clinical characteristics	
GOLD stages (II/III)	8/8
Length of hospital stay (days)	8.6 ± 4
Evaluation day (day)	4.5 ± 1.9
Use of oxygen therapy (%)	31.2
NIV use for a period ≥1 h/6 h (%)	50
Smoker/ex-smoker	10/6
Smoking load (pack years)	59.1 ± 23.4
Comorbidities	
Systemic arterial hypertension (%)	62.5
Diabetes (%)	18.8
Dyslipidemia (%)	31.3
Depression (%)	25
Hypothyroidism (%)	6.3
Obstructive sleep apnea syndrome (%)	6.3
Pulmonary function	
FEV_1_ (% predicted)	49.4 ± 9.9
FVC (% predicted)	77.3 ± 15.2
FEV_1_/FVC	0.5 ± 0.1
IC (liters)	1.7 ± 0.4
Questionnaires data	
MRC modified (score)	1.6 ± 1.3
BODE (score)	3.6 ± 2.2
CAT (score)	15 ± 5.3
CRQ—dyspnea (score)	3.3 ± 1
CRQ—fatigue (score)	3 ± 1.1
CRQ—emotional function (score)	4 ± 1.1
CRQ—self-control (score)	4.5 ± 1.5

Notes: Data are presented as mean ± SD. FEV₁/FVC, the ratio of FEV₁ to FVC; M = male; F = female; BMI, body mass index; Kg/m^2^ = kilograms per square meter; GOLD, global initiative for chronic obstructive lung disease; Y = yes; N = not; NIV, non-invasive ventilation; FEV_1_ (predicted%) = percentage of predicted for the forced expiratory volume in the first second; FVC (% predicted) = percentage of predicted for forced vital capacity; FVC/FEV_1_ = ratio of forced vital capacity to forced expiratory volume in the first second; IC, inspiratory capacity; Modified MRC, medical research council modified dyspnea scale; BODE = BODE, index (Body mass index, Airway Obstruction, Dyspnea, and Exercise capacity); CAT = COPD, assessment test; CRQ, chronic respiratory questionnaire.

**TABLE 2:** Performance average in first, second, and the best 6MWT and 6MST.

	6MWT	6MWT	6MWT
	(First test)	(Second test)	(Best test)
Performance (meters)	279.3 ± 90.8	300.6 ± 108.5	310 ± 96.2
Performance (%predicted)*	53.6 ± 15.2	59 ± 16.9	59.6 ± 16.7

	**6MST**	**6MST**	**6MST**
	**(First test)**	**(Second test)**	**(Best test)**
Performance (cycles)	141.8 ± 55.2	155.6 ± 62.9	159.8 ± 59.9

Notes: Data are presented as mean ± SD., 6MWT, 6-min walk test; 6MST, 6-min stepper test. * The derived equation for both genders was: 6MWT, distance = 356.658– (2,303 × age) + (36,648 × gender) + (1,704 × height) + (1,365 × ∆heart rate). When male gender = 1 and female gender = 0 (Britto et al., 2013).

**TABLE 3:** Physiological and perceptual parameters at rest (initial) and the end (final) of the best tests (6MWT and 6MST) performed.

6MWT (best test)	Initial	Final	Difference	Difference (%)
IC (liters)	1.7 ± 0.4	1.5 ± 0.4	−0.2	−11.8
SpO_2_ (%)	94.9 ± 2.8	92.4 ± 2.4	−2.4	−2.5
HR (bpm)	80.6 ± 12.3	96.6 ± 18.7	15.9	19.7
RR (bpm)	19.1 ± 3.6	21.9 ± 4.4	2.8	14.7
SBP (mmHg)	127.2 ± 7.7	143.8 ± 10.9	16.6	13.1
DBP (mmHg)	80.9 ± 8.4	83.4 ± 9.1	2.5	3.1
Borg Dyspnea	0.6 ± 1.1	1.1 ± 1.6	0.5	83.3
Borg Fatigue	0.4 ± 0.8	1.5 ± 1.7	1.1	275
**6MST (best test)**	**Initial**	**Final**	**Difference**	**Difference (%)**
IC (liters)	1.7 ± 0.4	1.5 ± 0.4	−0.2	−11.8
SpO_2_ (%)	94.3 ± 3	94.6 ± 3.7	0.3	0.3
HR (bpm)	84.6 ± 16.3	110.2 ± 20	25.6	30.3
RR (bpm)	18.9 ± 3.6	24.1 ± 5.6	5.3	28
SBP (mmHg)	121.6 ± 10.8	147.5 ± 23.2	25.9	21.3
DBP (mmHg)	72.8 ± 9.1	82.5 ± 14.6	9.7	13.3
Borg Dyspnea	0.1 ± 0.5	2.9 ± 2.9	2.8	2,900
Borg Fatigue	0.4 ± 1	5.1 ± 2.5	4.7	1,175

Notes: Data are presented as mean ± SD., 6MWT, 6-min walk test; 6MST, 6-min stepper test; IC, inspiratory capacity; SpO2, oxygen saturation; HR, heart rate; RR, respiratory rate; SBP, systolic blood pressure; DBP, diastolic blood pressure.

## Concurrent Validity

### Relationship of Performance on 6MST With Performance on 6MWT

**FIGURE 3:**
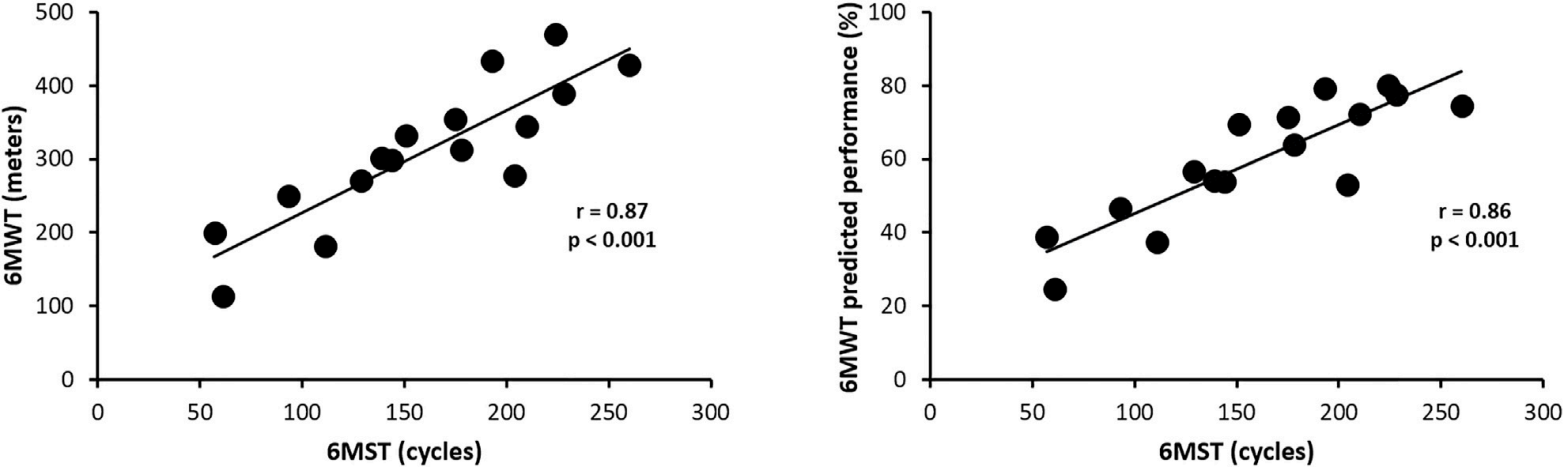
Correlation of performances in 6MST and 6MWT in absolute values and predicted values. Abbreviations: 6MST, 6-min stepper test; 6MWT, 6-min walk test.

The performance of individuals in the 6MST showed a strong correlation both with the absolute value (r = 0.87; *p* < 0.001) and with the predicted value (r = 0.86; *p* < 0.001) in the 6MWT (Figure 3).

### Relationship of Performance on 6MST With Dynamic Hyperinflation (Absolute and Percentage)

**FIGURE 4:**
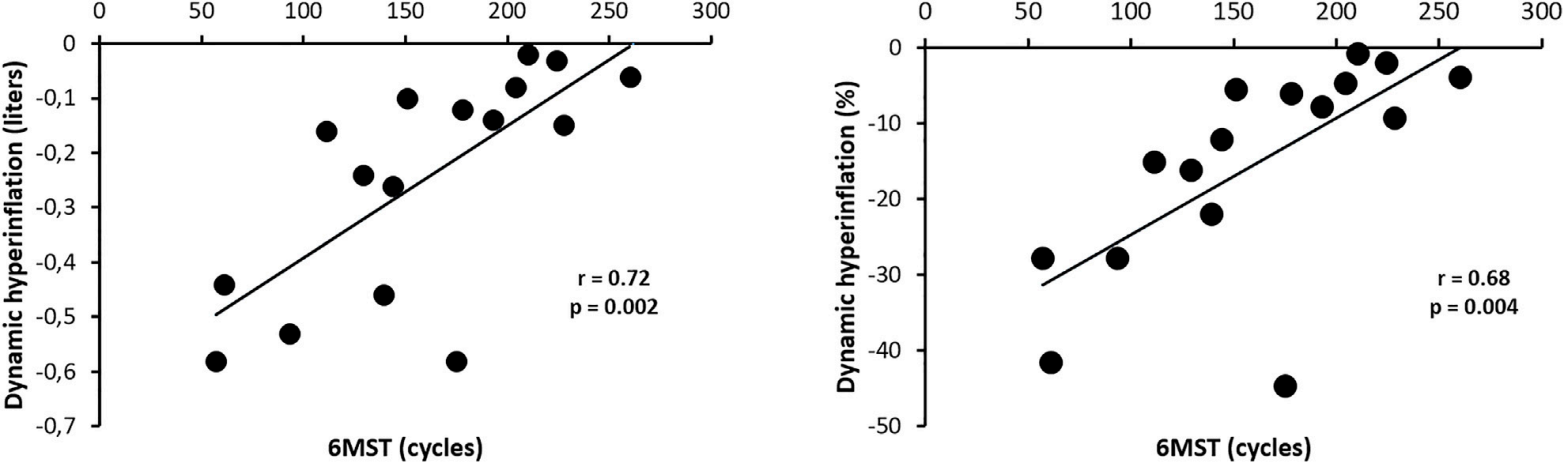
Correlation of 6MST performance with the dynamic hyperinflation in absolute values and percentage. Abbreviations: 6MST, 6-min stepper test.

There was a strong statistically significant correlation between 6MST performance and dynamic hyperinflation (DH) development in absolute values (r = 0.72; *p* = 0.002), and a statistically significant moderate correlation between 6MST performance and DH development in percentage values (r = 0.68; *p* = 0.004) (Figure 4).

### Relationship of Performance on 6MST With Questionnaires and Specific Indexes Associated With Lung Disease (Modified MRC, BODE, CAT, and CRQ)

**FIGURE 5:**
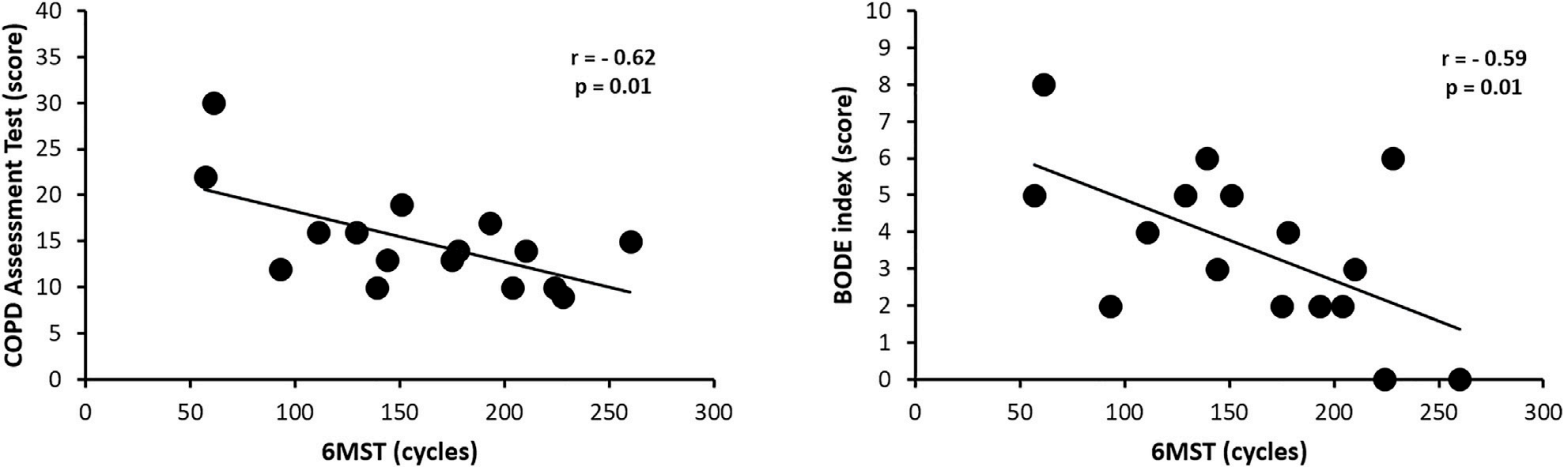
Correlation of 6MST performance with the COPD Assessment Test and the BODE index. Abbreviations: 6MST, 6-min stepper test.

There was a statistically significant moderate correlation between performance in 6MST and the score achieved in CAT (r = 0.62; *p* = 0.01) and BODE (r = 0.59; *p* = 0.01) (Figure 5). There was no statistically significant correlation between performance in 6MST and the score achieved on the modified MRC scale (*p* = 0.11), as well as in CRQ, in all domains: dyspnea (*p* = 0.19), fatigue (*p* = 0.19), emotional function (*p* = 0.96) and self-control (*p* = 0.30).

### Relationship of Performance on 6MST With Spirometric Variables (FEV₁, FEV₁/FVC, FVC, and Baseline IC)

There was no statistically significant correlation between 6MST performance and FEV₁ (*p* = 0.29), FEV₁/FVC (*p* = 0.50), FVC (*p* = 0.33) and baseline IC (*p* = 0.68).

### Relationship of Performance on 6MST with Variations in Vital Signs (Oxygen Pulse Saturation, HR, Systolic and Diastolic Blood Pressure, and RR) and the Perception of Dyspnea and Fatigue of Lower Limbs Before and After Testing

There was no statistically significant correlation between performance in 6MST and variations in both absolute and percentage of oxygen pulse saturation (*p* = 0.79 and 0.78, respectively), HR (*p* = 0.12 and 0.09, respectively), systolic blood pressure (*p* = 0.27 and 0.31, respectively), diastolic blood pressure (*p* = 0.55 and 0.46, respectively), RR (*p* = 0.10 and 0.33, respectively), and the absolute value of perception of dyspnea (*p* = 0.65) and lower limb fatigue (*p* = 0.97).

### Relationship Between the Variation's Magnitude of Vital Signs (Oxygen Pulse Saturation, HR, Systolic and Diastolic Blood Pressure, and RR) and Perception of Dyspnea and Fatigue of Lower Limbs fromRest During the 6MST and 6MWT

There was a statistically significant strong correlation between the magnitude of variation of respiratory rate (r = 0.77; *p* < 0.001) and moderate correlation between the magnitude of variation of perception of dyspnea (r = 0.50; *p* = 0.04) from rest during the 6MST and 6MWT. There was no statistically significant correlation between the magnitude of variation of IC (*p* = 0.17), oxygen pulse saturation (*p* = 0.34), HR (*p* = 0.14), systolic blood pressure (*p* = 0.06), diastolic blood pressure (*p* = 0.07), and the perception of lower limb fatigue (*p* = 0.8) from rest during the 6MST and 6MWT.

### Repeatability

The average performance on the first 6MST performed was 141.81 cycles, and on the second 6MST, it was 155.63 cycles, which means a difference of 13.82 cycles, with no statistical significance (*p* = 0.0575).

## Discussion

The current study aimed to verify the validity of 6MST for the assessment of functional capacity in hospitalized patients with AECOPD. Our results demonstrated the concurrent validation of 6MST with the 6MWT to evaluate functional capacity in this setting. A pioneering study of 6MST already suggested the concurrent validation of the test concerning the 6MWT; however, the population of this study was composed of individuals with COPD in a phase of clinical stability (Borel et al., 2010). Also, in this population of patients with stable COPD, enrolled in a pulmonary rehabilitation program, the concurrent validation of 6MST with the 6MWT was demonstrated by correlation, in addition to the distance covered in the 6MWT, both power and oxygen consumption at maximum effort (Wallaert et al., 2016). Besides that, other authors found a significant correlation between performance in 6MST with the distance covered in the 6MWT and oxygen consumption in frail elderly individuals (Jones et al., 2017), in a sample with a mean age of 71.2 years, a value close to the average age of the sample of our study (69.38 years).

Likewise, the present study suggested a correlation through the 6MST performance with the ventilatory responses (absolute value and percentage of DH), the degree of severity and mortality risk of the individual with COPD (BODE), and the impact of the disease on quality of life (CAT). Our results agree with a previous study (Marin et al., 2001), which also showed the development of DH in patients with COPD when they performed the 6MWT. These authors also found a statistically significant correlation between the final IC and performance on the 6MWT. Another study (Satake et al., 2015), in addition to correlating the development of DH with the distance covered in the 6MWT in individuals with COPD, also indicated a significant negative correlation between the decrease in IC and the increase in dyspnea after the 6MWT. To our knowledge, no study has analyzed the correlation between DH and 6MST. Regarding the degree of severity and mortality risk associated with COPD (BODE), the results of the present study suggest that the greater the impairment of this index, the worse the functional capacity in patients hospitalized for acute exacerbation of COPD. Although there are no studies directly correlating BODE with 6MST, previous studies have shown a significant correlation with other tests to assess functional capacity in individuals with COPD, such as the 6MWT and step test. As demonstrated in a previous study (Regueiro et al., 2009), the performance on the 6MWT of individuals with COPD showed a strong negative correlation with BODE. When correlated with the step test, another study demonstrated that there was a weak negative correlation between the performance in this test and the degree of impairment in the BODE index (Pessoa et al., 2014). The results of the present study indicate that the greater the impairment of the impact of COPD on quality of life (CAT), the worse the functional capacity in patients hospitalized for acute exacerbation of COPD. Although there are no studies directly correlating CAT with 6MST, previous studies have shown a significant correlation of the score with other tests for assessing functional capacity in individuals with COPD, such as the 6MWT and the 6-min pegboard and ring test. A previous study (Silva et al., 2013) had already demonstrated a significant negative correlation between CAT and the distance covered in the 6MWT, validating this questionnaire in the population of individuals with COPD. Also, another study (Jones et al., 2012) had already found a weak correlation between CAT and the performance of the 6MWT to assess responsiveness to a pulmonary rehabilitation program. A recent study also assessed functional capacity in hospitalized patients with exacerbated COPD, but in activities with the upper limbs (6-min pegboard and ring test), and demonstrated a moderate correlation of the test with CAT (Felisberto et al., 2018).

About the risk of a floor effect, is also important to note that, based on the percentage of patients in the sample who were able to adequately perform the 6MST (all patients were able to perform the test, with no dropout or the presence of important clinical alterations that required interruption of its execution) and evaluating the strong correlation between the 6MST and the 6MWT, a test that does not have a floor effect in this population, we were able to demonstrate the removal of the floor effect of the 6MWT in patients hospitalized for an acute exacerbation of COPD. This allows the 6MST to be used to assess the functional capacity of patients in the hospital setting, facilitating its use in situations such as the planning of individually designed therapeutic strategies and the optimization of exercise prescription, enabling a follow-up of their physical improvement, as well as the monitoring the evolution of the functionality and clinical condition in hospitalized patients with acute exacerbation of COPD.

Regarding repeatability, in our study, the average number of cycles performed in the second test was greater than the average number of cycles performed in the first test (an approximate difference of 14 cycles), but it was not considered statistically significant (*p* = 0.0575). Despite not having the minimum clinically important difference of 20 cycles suggested by a previous study (Pichon et al., 2015), a factor that may justify this result is probably the sample number, which was calculated to answer the main objective of the study - the concurrent validity of 6MST concerning the 6MWT to assess functional capacity in individuals with COPD. When calculating the number of sample to answer the specific repeatability objective that presented an 80% power, the estimated necessary number was 49 individuals. Although we are not able to conclude, our results corroborate previous studies (Borel et al., 2010; Coquart et al., 2014), which found the average performance higher in the second 6MST performed compared to the first 6MST. These authors suggest as justifications for this event (average of higher results in the second test), the possible learning effect in the performance of the second test, as well as the possibility of technical peculiarities of the stepper, such as heating the hydraulic shock absorbers of the device, causing less resistance to effort and influencing performance.

The present study has some limitations. Despite having reached the sample size calculation, as described in the statistical analysis, one of the limitations is the fact that this study was carried out in a single center and, therefore, had a reduced number of individuals composing the sample, which could influence the extrapolation of the findings to other hospitals. Likewise, as described earlier, as we did not calculate the sample specifically for the repeatability analysis, our data on this aspect could not provide a more definitive answer. Another limitation is that we did not assess the patients’ inspiratory capacity during the test, minute by minute, so that we could more closely monitor the development of DH.

## Conclusion

It could be concluded that 6MST is a valid test for evaluating functional capacity in hospitalized patients with exacerbated COPD.

## Data Availability

The raw data supporting the conclusion of this article will be made available by the authors, without undue reservation.

## Abbreviations

COPD, chronic obstructive pulmonary disease; 6MST, six-minute stepper test; AECOPD, acute exacerbation of COPD; CRQ, Chronic Respiratory Questionnaire; MMRC, Modified Medical Research Council Dyspnea Scale; CAT, COPD Assessment Test; 6MWT, six-minute walk test; COSMIN, COnsensus-based Standards for the selection of health Measurement INstruments; BMI, body mass index; FVC, forced vital capacity; FEV₁, forced expiratory volume in the first second; VC, vital capacity; IC, inspiratory capacity; ATS, American Thoracic Society; ERS, European Respiratory Society; DH, dynamic hyperinflation; RR, respiratory rate; HR, heart rate; SD, standard deviation.
